# Serum and Urine Biomarkers Related to Kidney Fibrosis Predict Kidney Outcome in Czech Patients with IgA Nephropathy

**DOI:** 10.3390/ijms24032064

**Published:** 2023-01-20

**Authors:** Michaela Neprasova, Dita Maixnerova, Nadja Sparding, Federica Genovese, Morten Asser Karsdal, Helena Koprivova, Marek Kollar, Miloslav Suchanek, Zdenka Hruskova, Vladimir Tesar

**Affiliations:** 1 Department of Nephrology, 1st Faculty of Medicine and General University Hospital, Charles University, 128 08 Prague, Czech Republic; 2 Nordic Bioscience, 2730 Herlev, Denmark; 3 Institute of Medical Biochemistry and Laboratory Diagnostics, 1st Faculty of Medicine and General University Hospital, Charles University, 128 08 Prague, Czech Republic; 4 Department of Clinical and Transplant Pathology, Institute of Clinical and Experimental Medicine, 140 21 Prague, Czech Republic; 5 Consultant of Chemometrics, 140 00 Prague, Czech Republic

**Keywords:** IgA nephropathy, biomarkers, PRO-C6, PRO-C3, LG1M, C3M, fibrosis, extracellular matrix

## Abstract

We evaluated biomarkers related to kidney fibrosis for the outcome of patients with IgA nephropathy (IgAN). Clinical parameters (estimated glomerular filtration rate, hypertension, proteinuria) and histological findings were assessed in 134 patients with IgAN at the time of diagnosis and followed up prospectively (mean follow-up time, 56.5 months). We measured biomarkers of collagen and laminin turnover in serum and urine collected at the time of kidney biopsy using a novel enzyme-linked immunosorbent assay. Linear discriminant analysis and logistic regression models were used to predict the patient’s kidney outcome. Five serum and urine biomarkers of laminin and collagen turnover (sLG1M, sPRO-C3, sPRO-C6, uPRO-C6/Cr, uC3M/Cr) could significantly differentiae IgAN patients with a worse prognosis. Clinical parameters (glomerular filtration rate (GFR), proteinuria) distinguished patients at risk of IgAN progression with a specificity of 87.3% and a sensitivity of 45.2% (area under the curve-AUC 0.751). The addition of the biomarkers significantly increased the prognostic ability with a specificity of 85.1% and a sensitivity of 73.3% (AUC 0.905). We have identified three serum (sLG1M, sPRO-C3, sPRO-C6) and two urinary markers (uPRO-C6/Cr, u-C3M /Cr) that significantly improve the prognostic ability of markers of kidney function to identify an IgAN patient’s risk of progressing to ESKD.

## 1. Introduction

IgA nephropathy (IgAN) is the most common primary glomerulonephritis with severe prognosis, and will see 30–50 % of patients developing end stage kidney disease. The main hallmark of chronic kidney diseases (CKD) is kidney fibrosis, which is caused by an imbalance between extracellular matrix formation and degradation [1,2]. The extent of kidney fibrosis is the best predictor of progression of most kidney diseases [3]. However, biopsy remains the only diagnostic tool that allows the reliable estimate of the degree of kidney fibrosis. Kidney biopsy is an invasive procedure associated with potentially serious life-threatening risks (especially bleeding), which cannot be repeated several times during the course of the disease. The collected tissue sample may not be representative of the whole kidney and does not provide information on the dynamics of tissue changes. At the time of disease diagnosis, immunosuppressive treatment of IgAN is indicated on the basis of clinical parameters (especially the level of proteinuria) and histological changes present in the initial kidney biopsy. If the proteinuria increases during the course of the disease, the decision to re-initiate immunosuppressive therapy is often difficult, and the clinical findings may not reflect the degree of acute or chronic changes.

In our study we evaluated serum and urine biomarkers of extracellular matrix turnover in patients with IgAN. The biomarkers we evaluated in our study, such as sPRO-C6 (C-terminal pro-peptide of collagen type VI in serum), sPRO-C3 (N-terminal pro-peptide of collagen type III in serum), uPRO-C6/Cr (C-terminal pro-peptide of collagen type VI measured in urine normalized by urine creatinine), uPRO-C3/Cr (N-terminal pro-peptide of collagen type III measured in urine normalized by urine creatinine), serum LG1M (fragment of MMP-laminin γ 1degragation) and urinary C3M/creatinine (fragment of MMP-mediated degradation of collagen type III measured in urine normalized by urine creatinine) are related to kidney fibrosis, which has been proven and described previously in other publications. We hypothesized that markers related to kidney fibrosis might help to predict kidney outcome in patients with IgAN and subsequently lead to targeted treatment with respect to active or chronic lesions.

## 2. Results

**Figure 1:**
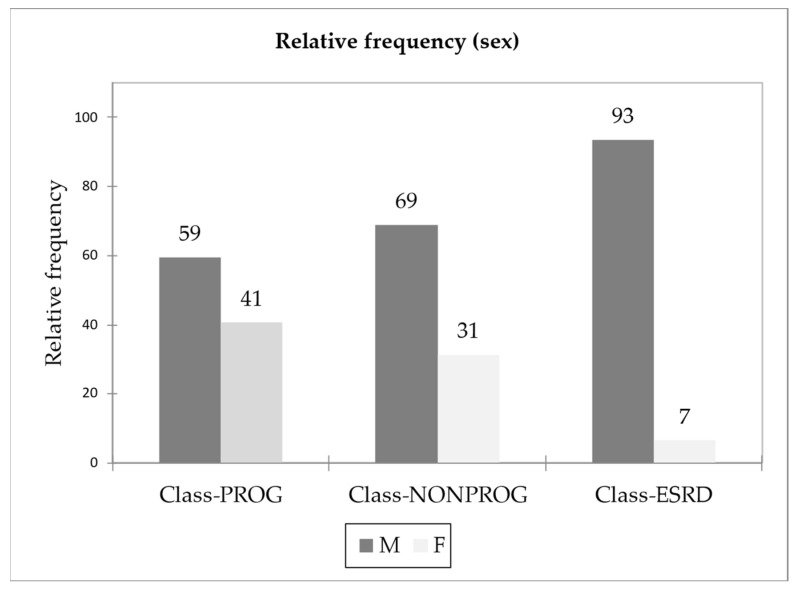
Gender distribution in NONPROG, PROG and ESKD groups.

We evaluated 134 Czech patients with biopsy-proven IgAN and a mean follow-up of 56.5 months. A total of 91 men with an average age of 45.9 years and 40 women with an average age of 42.4 years were examined in our cohort. All subjects were Caucasians. We divided the patients into three groups according to the changes of kidney function. The non-progressor group (NONPROG) included patients with a stable kidney function (mean decrease in the level of serum creatinine of 4% at the end of follow-up), the progressor group (PROG) included patients with a mean increase of serum creatinine at least of 35% at the end of the follow-up, and the end-stage kidney disease group (ESKD) included patients with a mean increase of the level of serum creatinine of 125% with the necessity of kidney replacement therapy by the end of the follow-up. The mean age of patients was 43.8 years for NONPROG, 48.8 years for PROG and 42.2 years for ESKD (Table 1). The representation of the examined subject in individual groups by gender is described in the Figure 1.

Basic statistical data of clinical and biochemical parameters at the time of biopsy are shown in Table 2, Table 3 and Table 4.

Classification according to progressive changes in creatinine (3 groups):

- NONPROG; PROG; ESKD: classification according to clustering
- NONPROG: average concentration of S-creatinine decreased by 4%
- PROG: average concentration of S-creatinine increased by 35%
- ESKD: average concentration of S-creatinine increased by 125%
- sLG1M—levels of a fragment of MMP-laminin γ 1degragation measured in serum at the time of biopsy (ng/mL)
- sPRO-C3—levels of the N-terminal pro-peptide of collagen type III measured in serum at the time of biopsy (ng/mL)
- sPRO-C6—levels of the C-terminal pro-peptide of collagen type VI in serum measured at the time of biopsy (ng/mL)
- uC3M/Cr—levels of a fragment of MMP-mediated degradation of collagen type III measured in urine (normalized by urine creatinine) at the time of biopsy (ng/mg)
- uPRO-C6/Cr—levels of the C-terminal pro-peptide of collagen type VI measured in urine (normalized by urine creatinine) at the time of biopsy (ng/mg)
- uPRO-C3/Cr—levels of N-terminal pro-peptide of collagen type III measured in urine (normalized by urine creatinine) at the time of biopsy (ng/mg)
- eGFR—estimated glomerular filtration rate at the time of biopsy (ml/min)
- PU—proteinuria (g/24 h)
- CRP—C-reactive protein (mg/L)
- Creatinine in serum (µmol/L)

We evaluated kidney fibrosis in the time of biopsy in our cohort. There were 113 samples in our cohort that met the criteria for histological evaluation. At the time of biopsy, the median of fibrosis in the NONPROG group was 15%, the median of fibrosis in the PROG group was 40% and the median of the fibrosis in the ESKD group was 50%. The difference between NONPROG, PROG and ESKD groups was confirmed as statistically significant (*p* value was 0.001) (Table 5).

We evaluated clinical parameters and biochemical markers and their ability to differentiate patients according to potential progression of kidney insufficiency. Using the Kruskal–Wallis test, we determined clinical parameters and biochemical markers that correlated with the probability of kidney failure in our cohort. Biomarkers and clinical parameters with a p-value less than 0.0001 were able to differentiate patients with a severe course of the disease with statistical significance. These markers allowed us to identify patients with a worse prognosis for kidney survival. Biochemical markers sLG1M, sPRO-C3, sPRO-C6, uC3M/Cr, uPRO-C6/Cr and clinical values of proteinuria, creatinine and eGFR were the biomarkers that could significantly separate patients into PROG+ESKD and NONPROG (Table 6).

Patients in our cohort who were in the ESKD group already had severe renal insufficiency at the time of biopsy, while some already met the parameters of kidney failure at the time of biopsy. These patients would not benefit from immunosuppressive treatment, therefore we excluded the ESKD group from the next measurements. We focused on identifying patients with a worsening kidney prognosis during the follow-up (PROG); these patients may benefit significantly from immunosuppressive treatment. We evaluated the prognostic potential of kidney function at the baseline for the risk of kidney outcome (differentiation of PROG and NONPROG). We tried to develop several prediction models, always using one variable (clinical parameter or biomarker), but sensitivity and specificity of individual clinical parameters and biomarkers for differentiating the PROG and NONPROG groups were not sufficient for any of those models. (Table 7).

**Figure 2:**
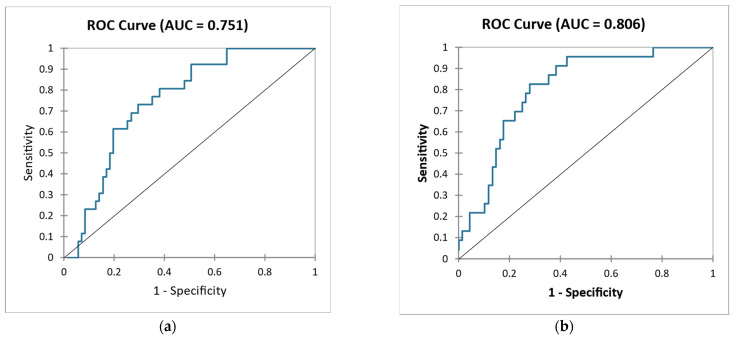
ROC curve—Differentiation of NONPROG and PROG using combination of clinical parameters eGFR, proteinuria (**a**) and using combination of all biomarkers (sLG1M, sPRO–C3, sPRO–C6, uPRO–C3/Cr, uPRO–C6/Cr, uC3M/Cr) (**b**).

Using a combination of all biomarkers, we achieved an improvement in the prediction of kidney outcome (sensitivity 50.0%, specificity 77.8%, AUC 0.806) compared to clinical parameters—eGFR and proteinuria (sensitivity 45.2%, specificity 87.3%, AUC 0.751). High sensitivity corresponds in our model to a good ranking of PROG patients and high specificity corresponds to a good ranking of NONPROG patients. (Figure 2).

**Figure 3:**
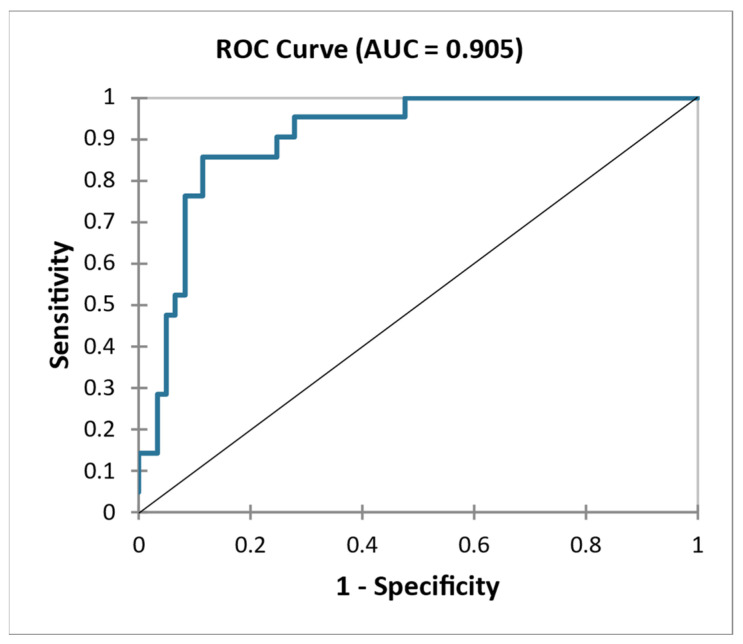
ROC curve—Differentiation of NONPROG and PROG using combination eGFR, proteinuria, sLG1M, sPRO–C3, sPRO–C6, uC3/Cr, uPRO–C3/Cr, uPRO–C6/Cr.

We built a model including the serum and urine biomarkers sLG1M, sPRO-C3, uPRO-C3/Cr, sPRO-C6, uPRO-C6/Cr, uC3M/Cr and eGFR and proteinuria. The addition of serum and urine biomarkers of extracellular matrix remodeling significantly improved the discriminatory ability of the markers of kidney function. The model had an accuracy of 82.9%, and the area under curve (AUC) was 0.905. (Figure 3).

**Figure 4:**
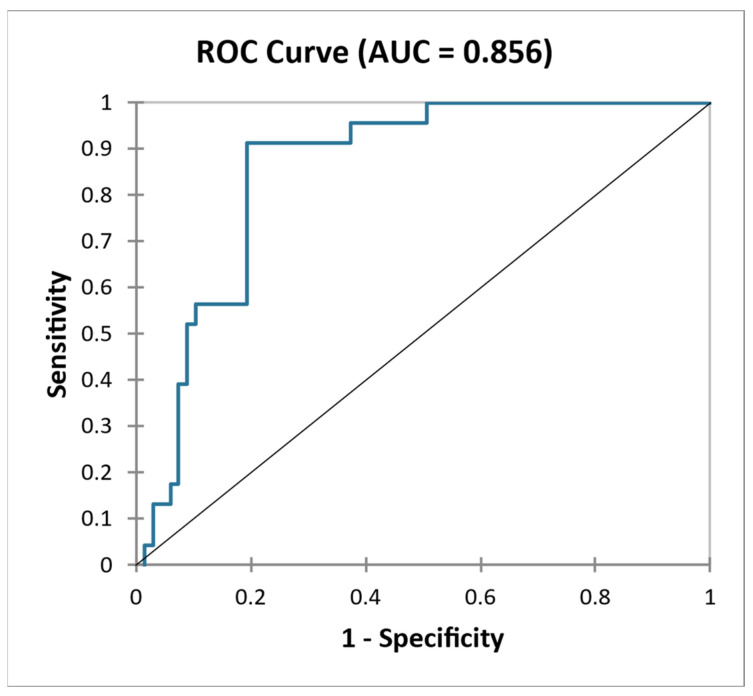
ROC curve—Differentiation of NONPROG and PROG with use of combination eGFR, proteinuria, MEST.

We then evaluated a model combining the markers of kidney function (eGFR), proteinuria and histological findings (MEST). The model had sensitivity of 35.9% and specificity of 100.0%, (AUC = 0.856) (Figure 4), showing that the addition of a non-invasive biomarker of extracellular matrix remodeling to markers of kidney function provided superior discriminatory power than adding histological biomarkers.

**Figure 5:**
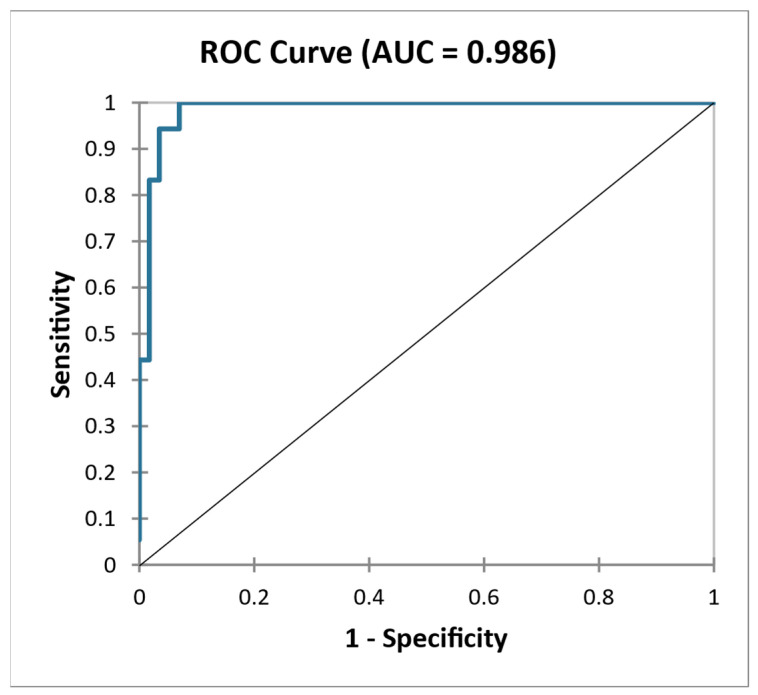
ROC curve—Differentiation of NONPROG and PROG using combination eGFR, proteinuria, MEST, sLG1M, sPRO–C3, sPRO–C6, uC3/Cr, uPRO–C3/Cr, uPRO–C6/Cr.

Adding histological findings (MEST) to the model, including markers of kidney function and biomarkers of extracellular matrix remodeling, allowed us to obtain an almost perfect discrimination between PROG and NONPROG: sensitivity 56.3 %, specificity 100.0% (AUC = 0.986) (Figure 5).

## 3. Discussion

Formation and progression of interstitial kidney fibrosis is the most common pathway leading to loss of kidney function [1]. Presence of kidney fibrosis is apparent in histology even before the kidney function decreases [4] and it predicts the development of end-stage kidney diseases in patients with IgAN [5]. The Oxford classification suggests that the major predictor of prognosis IgAN is tubular atrophy and interstitial fibrosis [5]. The basement membrane collagen type IV as well as the collagens type I and III are the most represented in the kidney scar [6]. Elevated serum and urine levels of the amino terminal peptide of procollagen III were observed in patients with chronic kidney disease, compared to healthy controls [6]. Elevated urinary concentration levels of collagen type IV were associated with a decline of kidney function in patients with type 1 and type 2 diabetes [7,8]. Moreover, increased collagen type IV expression was described in chronic transplant nephropathy [9].

Laminins are glycoproteins containing an α, β, and γ chain, which are a major component of the basement membranes [10]. Laminin is a substrate of matrix metalloproteinases 9 (MMP-9), an enzyme involved in extracellular matrix remodulation and upregulation of kidney fibrosis [11]. Mutations of the laminin genes can lead to kidney diseases [12]. The laminin gamma-1 chain (LAMC1) is the most common chain of laminin, is found in laminin-1–4 and 6–11 and is present in every basement membrane except in the central nervous system and in the retina [13]. Some authors assume that the presence of LAMC1 fragments could reflect the structural conversion of glomerular and tubular basement membranes and are considered as possible markers of a fibrosis [14]. LG1M is a neo-epitope fragment of the laminin generated by MMP-9 [14], and is a marker of basement membrane remodeling [14]. In the study, the authors demonstrated that high serum levels of LG1M is correlated with the progression of kidney insufficiency and the development of ESKD [14]. They demonstrated an association of high levels of LG1M in the urine with the death of patients in the study [14]. They hypothesize that changes in the basement membrane remodeling may contribute to pathological processes that lead to mortality in CKD patients. [14]. The authors of the study observed an association of serum LG1M with CRP and FLC and demonstrated that both associations indicate adverse CKD outcomes. [14].

In our study, we did not confirm a correlation between serum LGM1 levels and poor kidney prognosis. A statistically significant ability to predict worsened kidney outcome was demonstrated using only the whole block of biomarkers (sLGM1, sPRO-C3, sPRO-C6, uC3M/Cr, uPRO-C6/Cr, uPRO-C3/Cr).

Collagen type III is synthesized as procollagen containing a pro-peptide extension at both ends of the molecule. Increased concentration of N-terminal type III collagen pro-peptide (PRO-C3) occurs in many clinical conditions, where there is disintegration or accumulation of connective tissue, for example fibroproliferative, hematologic and malignant diseases [15]. In a study, PRO-C3 was associated with liver fibrogenesis in patients with chronic hepatitis C [15]. Urinary markers of collagen type III turnover in a particular urinary PRO-C3/C3M ratio is a potential novel, non-invasive diagnostic and prognostic marker to specifically monitor extracellular matrix (ECM) remodeling in kidney fibrosis in patients with IgAN [4]. Patients with IgAN and kidney fibrosis have an altered collagen turnover, the formation markers (PRO-C3) are increased, and the degradation markers (C3M) are lower in later CKD stages [4]. 

In the study with 500 patients with kidney impairment, PRO-C3 (a marker of collagen type III formation) and C3M (a peptide of helical collagen type III degradation) were measured in serum and urine using competitive enzyme-linked immunosorbent assays (ELISA). Urinary levels of C3M were inversely and independently associated with CDK progression and development ESKD and serum levels of PRO-C3 were associated with a risk of death [16]. 

Using serum or urinary PRO-C3 alone, we were unable to identify patients with an increased risk of kidney failure with sufficient sensitivity. However, an association with impaired kidney prognosis was demonstrated using clinical parameters (proteinuria, eGFR) and serum and urine biomarkers (sLGM1, sPRO-C3, sPRO-C6, uC3/Cr, uPRO-C3/Cr, uPRO-C6/Cr). In this case, the sensitivity of identifying high-risk patients was even higher than when using MEST.

Alternative pathway complement activation can be demonstrated in patients with IgAN and Henoch–Schönlein purpura (HSP) [17]; in patients with IgAN, the complement activation was associated with more severe kidney disease [17]. Plasma levels of activated C3 can indicate disease activity and kidney outcome [17]. Increased CRP values were found in IgAN patients with progressing disease compared to patients with stable disease during long-term monitoring [18]. Correlation of serum C3M and CRP was observed in IgAN patients [4], while spiking levels of C3M was associated with increased CRP levels and lower kidney function in the study [4].

We hypothesized an association between CRP and the development of kidney insufficiency, but this relation could not be demonstrated.

The study with 78 patients with kidney graft failure due to chronic injury or rejection examined the association of specific extracellular matrix biomarkers with the function of allograft and chronic kidney disease stage. Urinary levels of C3M correlated with eGFR rate (eGFR; r = 0.58, *p* < 0.0001), with lower levels detected in the urine of patients with advanced CKD [19]. Plasma levels of PRO-C6, a marker for collagen type VI formation, significantly increased with disease progression and correlated with eGFR (r = −0.72, *p* < 0.0001) but plasma C3M and urinary PRO-C6 levels showed no correlation with kidney function. [12]. PRO-C6 has been proposed as a non-invasive early marker of kidney fibrosis [2]. We assumed that serum PRO-C6 levels would correlate with decreased kidney function in our cohort as well. We did not prove this relation with statistical significance in our cohort. However, by using serum and urinary PRO-C6 in the block of biomarkers, we were able to identify patients at risk of disease progression.

## 4. Materials and Methods

In the study, we followed 134 patients with biopsy-confirmed IgAN, with a mean follow-up time of 56.5 months. We assessed the estimated glomerular filtration rate (eGFR), proteinuria, blood pressure and presence of microscopic hematuria at the time of kidney biopsy and at the end of follow-up. The urine collection at the time of the biopsy, out of which the markers were identified, was a spot collection. The value of proteinuria, which was used for the statistical processing of the file, was from the 24-h collection at the time of biopsy and at the end of monitoring. PRO-C6 values have already been measured in 34 patients of our cohort and results have been published (Endotrophin, a collagen type VI-derived matrikine, reflects the degree of renal fibrosis in patients with IgA nephropathy and in patients with ANCA-associated vasculitis, N. Sparding et al., NDT, 25 May 2022). The biomarkers of laminin (LG1M), collagen type III (PRO-C3 and C3M) and collagen type IV (PRO-C6) were measured in serum and urine using enzyme-linked immunosorbent assays (ELISAs) developed and produced at Nordic Bioscience (Herlev, Denmark) according to the manufacturer’s instructions and as detailed in the references in the Table 8.

The LG1M assay used a monoclonal antibody (mAb) detecting the sequence LNRKYEQAKN corresponding to the cleavage site of MMP-9 in position 1241 of laminin γ1. The PRO-C3 assay used a mAb detecting the sequence PTGGQNYSP corresponding to the cleavage site by ADAMTS-2 of the N-terminal pro-peptide of collagen type III. The C3M assay used a mAb detecting the sequence KNGETGPQGP corresponding to the cleavage site of MMP-9 in position 610 of collagen type III. The PRO-C6 assay uses a mAb detecting the sequence KPGVISVMGT, corresponding to the last 10 amino acids of the collagen type VI α3 chain. The concentration of the urinary markers was divided by urinary creatinine to adjust for urine volume. Urinary creatinine was measured by the QuantiChrom Creatinine Assay Kit (BioAssay Systems, Hayward, CA, USA). We evaluated histological findings in kidney biopsy specimens from patients with IgAN [fibrosis—≤20% (class 1), ≤50% (class 2), >50% (class 3), MEST—C-score]. *p*-values were calculating using the Kruskal–Wallis test, while linear discriminant analysis was used to calculate areas under curves (AUC).

## 5. Conclusions

We demonstrated that serum and urine biomarkers related to kidney fibrosis such sPRO-C6, sPRO-C3, uPRO-C6/Cr, serum LG1M and urinary C3M/creatinine predicted kidney outcome of patients with IgAN in the cohort of Czech IgAN patients. Serum and urinary markers of fibrosis in combination with clinical parameters (eGFR, proteinuria) significantly increase the ability to identify patients at risk of progression of kidney insufficiency. Serum and urine fibrosis markers in our cohort reliably predict an unfavorable prognosis of the disease even without the use of histological parameters. The ability to predict a worsened prognosis of kidney disease using serum and urine biomarkers could help identify patients who should receive the immunosuppressive treatment even repeatedly during the course of the disease and thus avoid kidney re-biopsy. The results of our study are limited by the use a small file of patients of one ethnicity. In the future, it will be necessary to confirm our conclusions on larger files. In order to extend the clinical applicability of the findings it will also be necessary to expand the study to include research on patients of other ethnicities.

## Figures and Tables

**Table 1:** Age in different classes (years).

Statistic	NONPROG	PROG	ESKD
No. of observations	79	39	16
Median	42.0	49.5	44.0
Mean	43.8	48.8	42.2
Standard deviation	14.8	14.8	13.8

**Table 2:** Basic statistic of eGFR, proteinuria, serum creatinine and CRP at the time of biopsy.

Statistic	eGFR			Proteinuria			Creatinine			CRP		
	NONPROG	PROG	ESKD	NONPROG	PROG	ESKD	NONPROG	PROG	ESKD	NONPROG	PROG	ESKD
No. of observations	79	28	11	79	28	11	79	28	11	79	28	11
No. of missing values	5	2	0	5	1	0	2	1	0	6	1	0
Median	63.0	36.3	36.0	1.3	1.7	3.0	114.0	177.0	207.0	2.4	177.0	207.0
Mean	73.7	38.4	37.2	2.8	2.5	3.0	132.0	203.4	284.6	7.4	203.4	284.6
Standard deviation	48.3	18.5	25.6	3.3	2.9	1.4	68.1	111.6	226.3	18.4	111.6	226.3
Median of absolute deviation	27.0	12.3	16.8	1.0	0.8	1.0	36.0	40.0	82.0	1.4	40.0	82.0
Interquartile range	55.5	24.5	25.5	2.8	1.9	1.6	75.0	84.0	186.5	4.7	84.0	186.5

**Table 3:** Basic statistic of biochemical parameters at the time of biopsy (sLG1M, sPRO-C3, sPRO-6).

Statistic	sLG1M			sPRO-C3			sPRO-C6		
	NONPROG	PROG	ESKD	NONPROG	PROG	ESKD	NONPROG	PROG	ESKD
No. of observations	79	28	11	79	28	11	79	28	11
No. of missing values	0	0	0	0	0	0	0	5	5
Median	5.5	5.5	5.2	9.2	10.7	9.4	12.7	2.1	8.5
Mean	9.0	9.4	11.4	9.9	11.2	11.6	15.1	19.0	35.3
Standard deviation	9.1	7.8	11.3	4.3	3.1	6.2	9.0	46.3	47.2
Median of absolute deviation	1.9	1.9	1.6	2.2	1.4	1.4	3.1	0.9	6.9
Interquartile range	7.3	7.2	12.3	4.2	3.3	3.1	7.1	16.9	70.5

**Table 4:** Basic statistic of biochemical parameters at the time of biopsy (uC3M/Cr, uPRO-C3/Cr, uPRO-C6/Cr).

Statistic	uC3M/Cr			uPRO-C3/Cr			uPRO-C6/Cr		
	NONPROG	PROG	ESKD	NONPROG	PROG	ESKD	NONPROG	PROG	ESKD
No. of observations	79	28	11	79	28	11	79	28	11
No. of missing values	11	5	5	11	5	5	11	5	5
Median	60.9	36.1	39.3	3.3	3.7	5.0	1.7	2.1	8.5
Mean	64.9	41.0	45.9	4.2	6.2	5.0	5.7	19.0	35.3
Standard deviation	30.6	23.7	30.4	3.8	8.2	1.7	15.1	46.3	47.2
Median of absolute deviation	20.1	16.3	23.3	1.6	1.7	0.4	0.6	0.9	6.9
Interquartile range	39.3	30.0	48.2	3.5	3.5	0.6	1.2	16.9	70.5

**Table 5:** Classification according to the kidney fibrosis (%).

	N	Median
All	113	30
NONPROG	77	15
PROG	26	40
ESKD	10	50

**Table 6:** Kruskal–Wallis test–the comparison clinical and biochemical markers for PROG+ESKD and NONPROG.

Variable	*p*-Values
CRP	0.693
sLG1M	0.000
sPRO-C3	0.000
sPRO-C6	<0.0001
uC3M/Cr	<0.0001
uPRO-C6/Cr	<0.0001
uPRO-C3/Cr	0.071
Creatinine	<0.0001
PU	0.024
eGFR	<0.0001

**Table 7:** Individuals ROC curves for all parameters and sensitivity and/or specificity.

Bio	AUC	Sensitivity	Specificity	Correct Classification
uC3M/Cr	0.737	0.0%	74.7%	74.7%
Creatinine	0.735	50.0%	76.6%	74.1%
eGFR	0.722	42.5%	85.0%	68.0%
sPRO-C6	0.716	40.0%	75.3%	72.0%
PU	0.648	0.0%	73.3%	73.3%
uPRO-C6/Cr	0.630	60.0%	76.7%	75.8%
sPRO-C3	0.625	0.0%	73.8%	73.8%
sLG1M	0.531	0.0%	73.8%	73.8%
uPRO-C3/Cr	0.508	50.0%	75.9%	74.7%
MEST	0.828	28.1%	100.0%	36.0%

**Table 8:** Biomarkers measurement references.

Biomarker	Description	Measures	Serum	Urine	Reference
LG1M	A fragment of laminin released by MMP-9	Basement membrane degradation	✔		[14]
PRO-C3	Cleaved N-terminal pro-peptide of collagen type III	Interstitial matrix formation	✔		[20]
C3M	A fragment of type III collagen released by MMP-9	Interstitial matrix degradation		✔	[4]
PRO-C6	C-terminal pro-peptide of type VI collagen (α3 chain)	Interstitial matrix formation and endotrophin	✔	✔	[21]

## Data Availability

Not applicable.
